# Sensitive Marker Affected by All Systemic Abnormalities—troponine

**DOI:** 10.5152/eurasianjmed.2022.22291

**Published:** 2022-12-01

**Authors:** Gökhan Ceyhun

**Affiliations:** 1 Department of Cardiology, Atatürk University Faculty of Medicine, Erzurum, Turkey

**Keywords:** Troponin, non-cardiac, non-coronary

## Abstract

Whether due to myocardial infarction or not, we find troponin levels to be high when myocardial damage occurs. Elevated troponin may not always be a myocardial infarction caused by plaque rupture on the basis of classically known coronary thrombosis. In this sense, infarction types have been defined. In this review, we aimed to bring together the conditions that may directly or indirectly affect the myocardium, except for acute coronary syndromes.

**Main Points**

- Troponin is a highly sensitive biochemical marker for the diagnosis of acute coronary syndrome. Troponin elevation, which is frequently evaluated outside the cardiology clinic, can sometimes lead to complexity in terms of diagnosis and treatment.
- Factors that may directly or indirectly cause myocardial damage, except acute coronary syndromes that occur with classical atherosclerosis pathophysiology, were brought together.
- After exclusion of acute coronary syndrome, major conditions that may cause troponin elevation were brought together.

## Introduction

Troponin is the first biochemical marker that comes to mind when heart muscle damage is mentioned. It is used in many heart diseases, especially myocardial infarction. Troponin is a component of the contractile apparatus found in skeletal muscle and cardiac myocytes. Troponin proteins, together with calcium ions, regulate the interaction between actin and myosin filaments as part of the sliding filament mechanism in muscle contraction.^1^ Cardiac troponin (Ct) is a complex of 3 subunits. Of these, the cardiac specificity of Ctc is low since its isoform in skeletal muscle has a similar structure. Cti and Ctt, on the other hand, are produced by completely different genes and have different amino acid sequences compared to their isoforms in skeletal muscle, so their cardiac specificity is quite high. In recent years, a highly sensitive cardiac troponin T test has been developed to speed up the diagnosis. Thus, it is possible to detect very low values even in patients with subclinical acute coronary syndrome. Since this sensitivity is high in non-coronary diseases, it brings diagnostic confusion. In this review, we will try to talk about non-cardiac conditions that cause troponin elevation and are investigated in the literature.

### Exposure to Toxic Agents

Neurogenic, toxic, vascular, and hemodynamic disorders that may affect the contractility of the heart may directly or indirectly affect the heart, and this may first appear as troponin elevation. Many medical or external toxic conditions can lead to myocardial damage. For example, myocarditis and even injury-related acute heart failure have been reported after a scorpion sting.^2^ Significant myocardial damage and electrocardiogram (ECG) changes were observed in carbon monoxide poisoning, another toxic cause.^3-6^ Although rare, the effect of troponin on iron and fungal intoxication has been reported.^7,8^ Mild troponin elevations, which are elevated in myocardial damage caused by toxic agents, have generally been a marker of reversible damage.^9^

### Oncological Drugs

The increase in oncological cases has led to the widespread use of chemotherapy. Many chemotherapeutic agents are cardiotoxic, and it is necessary to determine the cardiological risk status during the treatment process.^10^ For this risk situation, a certain troponin cutoff value has been tried to be determined.^11^ Anthracycline-based chemotherapeutic agents used in the treatment of breast cancer are among the most important cardiotoxic drugs known. Serum troponin values were used as indicators of cardiac toxicity in breast cancer patients receiving epirubicin-based chemotherapy.^12^ Doxorubicin has serious cardiotoxic effects, and ramipril is protective from this effect.^13,14^ 5-Fluorourasil (5-FU) causes myocardial ischemia with coronary vasospasm and endothelial damage.^15^ In the study of Gelen et al, in addition to severe myocardial damage, ECG changes were observed in rats given 5-FU.^16^ Multitargeted kinase inhibitors targeting BCR-ABLs such as imatinib, bosutinib, and dasatinib can cause pleural and pericardial effusion and heart failure.^17^ Taxifolin is protective against these effects.^18^ The cardiotoxic effects of sunitinib with anti-angiogenesis effect and interferon-α used in the treatment of hepatitis b have been demonstrated.^19,20^ Use of vascular endothelial growth factor in ischemic tissues has shown benefit in patients with peripheral vascular disease.^21^ Vascular endothelial growth factor inhibitors are used in the treatment of many types of cancer, including hepatocellular carcinomas, and have many cardiovascular side effects.^22^ Ibrutinib, one of the Bruton tyrosine kinase inhibitors, is frequently used in lymphoid cancers known to cause heart failure and atrial fibrillation.^23^ Immunological agents used in cancer treatment should be discontinued in case of pericarditis, cardiac dysfunction, arrhythmias, and myocardial infarction.^24,25^

A group of non-malignant hematological diseases can lead to troponin elevation by affecting myocardial functions. Examples of these are hypereosinophilic syndrome, thrombotic thrombocytopenic purpura, and hemolytic uremic syndrome.^26,27^ Although not as much as chemotherapy in oncological treatments, radiotherapy given to the chest area, especially for breast and lung cancers, may cause myocardial damage.^28,29^

### Cerebrovascular Events

Cerebral hemorrhage may also cause myocardial damage by increasing intracranial pressure and affecting the sympathetic balance of the heart.^30^ High troponin values have been associated with mortality in ischemic stroke.^31-34^ Changes in myocardial function in patients in the early stage of acute ischemic stroke have been detected by echocardiography. Correlation was observed between the extent of infarction and some echocardiographic parameters.^35^

### Pulmonary Embolism

When defining massive and submassive pulmonary embolism, the patient’s blood pressure, right ventricular functions, and troponin values are determined.^36^ High troponin values in acute pulmonary embolism are used to determine the degree of impaired right ventricular function.^37^ Many troponin-based risk classifications have been established in normotensive patients with acute symptomatic pulmonary embolism.^38^ Kerget et al stated that Vascular endothelial growth factor (VEGF)-D could be an alternative to troponin and Nt-pro BNP in pulmonary embolism clinical risk scoring.^39^

### Hypertension

Hypertensive crisis is defined as a sudden rise in blood pressure above 180/120 mmHg and is associated with poor clinical outcomes. High troponin values observed in hypertensive crisis can be attributed to myocardial supply–demand mismatch or existing obstructive coronary artery disease. Increased troponin values have been associated with major adverse cardiac or cerebrovascular events.^40^ It is known that preeclampsia is a multisystem disease with capillary damage and vasospasm mediating kidney, placenta, and cerebral damage.^41^ Barton et al^42^ described the histopathological changes occurring in the myocardium in preeclampsia. Minimally high troponin levels can be detected in normal pregnancy, but this elevation is more pronounced in pregnant women who develop hypertension.^43^

### Infective Agents

Troponin levels are mostly high in systemic and local infections, especially in the patient group with sepsis.^44^ Viral infections are effective in the formation of myocarditis. Coronavirus disease-2019 (COVID-19) is a potentially fatal disease with main clinical manifestations ranging from mild respiratory distress to severe acute respiratory distress syndrome. Since coronavirus is a viral infection, it primarily affects the lungs, but the resulting inflammatory condition can also affect the heart. High-sensitivity cardiac troponin levels are elevated in a significant proportion of cases with COVID-19 infection. It has been reported that increase in cTn is observed in 17%-20% of patients and in >50% of patients who died.^45^ Troponin values can be used to predict the prognosis and extent of the disease.^46,47^

### Kidney Failure

Cardiovascular diseases are responsible for nearly half of the deaths in patients with end-stage renal disease. Over the past decade, several studies have emerged that demonstrated that elevated cardiac-specific troponins may predict mortality in patients with chronic renal failure without acute coronary syndrome.^48-51^ Although high troponin values are clinically asymptomatic, they are in a subgroup of chronic renal failure patients with poor survival and a high risk of cardiac death.^52^ Similarly, it has been stated that troponin values for early diagnosis may be beneficial before the increase in creatinine in acute renal failure.^53,54^

### Other Reasons

In addition to all these, except for acute coronary syndromes, blunt chest trauma, arrhythmias, heart failure, electric shock, aortic valve stenosis, gastrointestinal bleeding, cardioversion defibrillation, burns, overexertion, hypertensive crisis, infiltrative diseases, and troponin after ablations may still be high.^55,56^
